# Exploring bi-directional impacts of Lisdexamfetamine dimesylate on psychological comorbidities and quality of life in people with Binge Eating Disorder

**DOI:** 10.1186/s40337-024-01041-9

**Published:** 2024-06-13

**Authors:** Kristi R. Griffiths, Stephanie Boulet, Sarah Barakat, Stephen Touyz, Phillipa Hay, Sarah Maguire, Michael R. Kohn

**Affiliations:** 1 InsideOut Institute, Charles Perkins Centre, University of Sydney, Sydney Local Health District, Sydney, NSW 2006 Australia; 2 Brain Dynamics Centre, Westmead Institute for Medical Research, University of Sydney, Westmead, NSW Australia; 3 Clinical Psychology Unit, School of Psychology, University of Sydney, Sydney, Australia; 4 Translational Health Research Institute, School of Medicine, Western Sydney University, Sydney, Australia; 5 Centre for Research Into Adolescents’ Health (CRASH), University of Sydney, Sydney, Australia; 6 Adolescent and Young Adult Medicine, Westmead Hospital, Sydney, NSW Australia

**Keywords:** Mental Health, ADHD, Anxiety, Depression, Psychopharmacology, Pharmacotherapy, Medication

## Abstract

**Background:**

Lisdexamfetamine dimesylate (LDX) has demonstrated safety and efficacy for treatment of Binge Eating Disorder (BED). However, to date, trials have not included participants with co-occurring psychiatric disorders. This study explores how LDX affects eating disorder psychopathology, symptoms of common psychiatric comorbidities of BED (ADHD, depression, anxiety), and psychological quality of life, in people with moderate to severe BED.

**Methods:**

These are secondary analyses of an open-label LDX trial conducted in 41 adults (18–40 years) over eight-weeks. Participants received LDX titrated to 50 or 70 mg. Clinical assessments and self-report questionnaires were conducted at baseline and 8-week follow-up.

**Results:**

Eating disorder psychopathology and psychological quality of life improved after 8-weeks of LDX. No significant group-level changes in depression, anxiety or ADHD severity scores were observed. However, the majority within the small subsets with elevated depression and ADHD symptoms experienced reduced depressive and inattentive symptom severity, respectively.

**Conclusions:**

We provide proof-of-concept evidence that LDX may provide broader psychological benefits to individuals with BED, beyond reducing their BE frequency. Effects of LDX on anxiety should be monitored closely by clinicians. Early indications suggest that LDX may be effectively used in people with BED, with and without co-occurring psychiatric conditions, however tolerability may be lower in highly complex cases.

*Trial registration*: Australian and New Zealand Clinical Trials Registry (anzctr.org.au) #ACTRN12618000623291.

**Supplementary Information:**

The online version contains supplementary material available at 10.1186/s40337-024-01041-9.

## Abstract (plain language summary)

Lisdexamfetamine dimesylate (LDX) has been shown to reduce binge eating frequency among those with Binge Eating Disorder (BED). However, little is known about how LDX affects symptoms of common co-occurring conditions (ADHD, depression, anxiety) and mental health more broadly. In this study, 41 people with BED received an 8-week course of LDX and their symptoms were monitored before and after treatment. Overall, people experienced a robust improvement in eating disorder psychopathology and psychological quality of life. For those with higher levels of depression and ADHD, LDX had the additional benefit of improving depressive symptoms and inattentive symptom severity, respectively. The effect of LDX on anxiety symptoms appears to be more complex, with an equal proportion of people experiencing a decrease or an increase in anxiety over the course of treatment. Those who experienced reductions in anxiety during treatment tended to have greater concurrent reductions in binge eating frequency. This study provides preliminary evidence that for people with BED, LDX may be effective at improving co-occurring symptoms of eating disorder psychopathology and psychological well-being, and potentially ADHD and depression symptoms when present at an elevated level. More research is needed among a larger sample to verify these findings.

**Supplementary Information:**

The online version contains supplementary material available at 10.1186/s40337-024-01041-9.

## Background

Binge Eating Disorder (BED) is a serious psychiatric condition that is characterised by recurrent episodes of binge eating and associated with significant psychological distress [1]. BED has a 12-month weighted mean prevalence of 1.4% (0.5–3%) for women and 0.6% (0–1.2%) for men [2] and accounts for approximately 40% of the global burden from eating disorders [1]. Individuals with BED exhibit high rates of psychiatric comorbidities, with 93.8% of a US-based adult sample found to meet criteria for at least one additional psychiatric disorder [3]. Among the most common mental illnesses to co-occur with BED are lifetime affective disorders (69.9%), anxiety disorders (59%) and post-traumatic stress disorder (31.6%). Disorders characterised by poor impulse control, such as Attention Deficit Hyperactivity Disorder (ADHD) and alcohol use disorder, also commonly co-occur with BED [4]. Comorbidities among people with eating disorders are associated with increased symptom severity, as well as poorer functioning and outcomes [5], making an already burdensome illness more challenging to treat.

Lisdexamfetamine dimesylate (LDX) (Vyvanse) is a pro-drug of D-amphetamine that is approved by the United States Food and Drug Administration (FDA) and similarly by the Therapeutic Goods Administration (TGA) in Australia, as the first, and currently only, drug for the treatment of moderate to severe Binge Eating Disorder. Placebo-controlled clinical trials have demonstrated safety and efficacy in reducing binge eating frequency [6–9], as well as reducing impulsivity [10, 11] and enhancing sustained attention [12].

Of note, LDX is also approved to treat symptoms of impulsivity and inattention in ADHD. This would suggest that there are commonalities in the pathophysiology of BED and ADHD that may both be addressed through the pharmacological action of LDX [13], however there is currently no published evidence to support this assumption. There is less clarity on how LDX may impact other common co-occurring illnesses. LDX has been examined as an adjunct therapy for individuals with major depressive disorder (MDD) who have failed to respond to an antidepressant. It is theorised that the inhibition of dopamine and noradrenaline reuptake may improve common symptoms of fatigue and cognitive dysfunction [14]. While there is limited data (four studies), a meta-analysis found that when used as an antidepressant adjunct, LDX produced a small effect in improving depressive symptoms that approached trend-level significance [14]. However, the generalisability of these effects of LDX on ADHD and MDD to people with co-occurring BED is unknown.

Despite the high rates of psychiatric comorbidity experienced by individuals with BED, to date, all clinical trials examining the efficacy and tolerability of LDX in BED have specifically excluded participants with Axis I or II psychiatric disorders [6, 15]. As such, there is a large knowledge gap on how LDX impacts symptoms of commonly co-occurring psychiatric illnesses, and whether changes in these symptoms are related to concurrent changes in BE frequency. Furthermore, it remains to be determined as to whether specific co-occurring conditions may reduce the clinical efficacy of LDX. Given that co-occurring psychiatric disorders are the norm among people with BED, symptoms of co-occurring conditions should be considered in treatment, as part of an overall picture of global wellbeing.

This study aims to explore how LDX affects eating disorder psychopathology, symptoms of common psychiatric comorbidities of BED (ADHD, depression, anxiety) and overall psychological quality of life in people with moderate to severe BED. It further aims to determine whether there are any associations between changes in these measures and changes in BE frequency. Finally, this study aims to determine whether there is a specific clinical profile for individuals who will best tolerate LDX and experience the best efficacy. By improving our understanding of how LDX effects not only eating disorder symptomology, but also mental health more broadly, the overarching aim is to provide important information for guiding clinicians in their treatment planning for BED.

## Methods

### Study design

This paper presents a secondary analysis of an open-label trial of LDX, previously described in a trial protocol paper [16]. Participants with moderate to severe BED were recruited between April 2018 and January 2021 at the Westmead Institute for Medical Research and Westmead Hospital, Australia [16], and prescribed LDX over an eight-week course, titrated to 50 or 70 mg. Clinical assessments and self-report questionnaires were conducted at baseline and medicated eight-week follow-up.

Ethics approval was granted by the Western Sydney Local Health District Human Research Ethics Committee and the trial was registered with the Australian and New Zealand Clinical Trials Registry (anzctr.org.au) #ACTRN12618000623291. Informed written consent was provided by participants.

### Participants

Forty-six participants (aged 18–40 years) with a primary diagnosis of moderate to severe BED were enrolled into the study. Participants were referred from participating clinicians or self-referred from online Facebook advertisements and a clinical trial recruitment agency, TrialFacts. Participants were required to meet criteria for BED as per the Diagnostic and Statistical Manual of Mental Disorders, Fifth Edition (DSM-5), which was verified using the eating disorders module of the Structured Clinical Interview for DSM-5. Participants were additionally required to have reported at least three days of binge eating per week in the past month, as well as a minimum score of 4 (“moderately ill”) on the CGI-S (Clinical Global Impressions-Severity) [17]. For inclusion, participants were required to have a body mass index (BMI) between 20 and 45 kg/m and medical approval to start LDX. Those with a current diagnosis of bulimia nervosa, anorexia nervosa, psychosis, mania and substance dependence; history of physical brain injury; psychostimulant use in the past six months; or current therapy with antipsychotics, noradrenaline reuptake inhibitors or monoamine oxidase inhibitors were excluded ([16] for full list of inclusion and exclusion criteria). These exclusion criteria were primarily selected to ensure a correct eating disorders diagnosis, and as contraindications to being treated with LDX. Participants were allowed to remain on any other existing medications deemed safe by the prescribing clinician, provided these treatments remained unchanged across the course of the trial. Co-occurring psychiatric and neurodevelopmental conditions were identified with the MINI International Neuropsychiatric Interview for DSM-5 [18].

### Treatment

Participants with BED completed an eight-week course of LDX, commencing with 30 mg/day of LDX, as per clinical practice [18, 19]. Participants were advised to take their medication each morning at the same time and were given self-monitoring sheets to track compliance.

Participants experiencing no abnormal cardiovascular changes at the two-week mark, as assessed by the study clinician, increased their dose to 50 mg/day. Clinical judgement regarding the experienced side effects and responsiveness to the medication at the four-week assessment was used to determine whether participants continued at 50 mg/day or increased to 70 mg/day. Participants maintained this dosage for the remaining four weeks of the trial. Completed self-monitoring sheets and unused tablets were returned at the week eight assessment.

In the case of protocol violations where participants were unable to attend their time two session at exactly eight weeks post baseline, participants either received additional medication to continue until the time two booking (for longer-term delays) or were asked to save their last tablet for the day of their time two scan (for short-term delays).

### Measures

Consistent with the seminal trials of LDX in BED [6, 11], the primary outcome measure for this clinical trial was BE frequency (days per week), which was assessed using clinical interviews at baseline and week eight.

#### Eating disorder psychopathology

The Eating Disorders Examination Questionnaire (EDE-Q) [19] is a 28-item self-report tool that measures eating disorder psychopathology over the preceding 28 days. It comprises of a global scale and four subscales related to restraint, eating concern, shape concern, and weight concern. All scales have a score range 0–6, with global scores above 2.3 shown to differentiate eating disorder cases from noncases [20]. The EDE-Q has been demonstrated to have sufficient concurrent and criterion validity [19] and reliability [21].

#### Depression/anxiety severity

The Hamilton Depression Rating Scale (HAM-D) measures the severity of depressive symptoms experienced over the past seven days [22]. The 17-item scale is clinician-administered and has good reliability [23] for the assessment of depression. A score of 0–7 (out of 52) is indicative of no depression; 8–16 mild depression; 17–23 moderate depression; and ≥ 24 severe depression [24].

The Hamilton Rating Scale for Anxiety (HAM-A) is a 14-item clinician-administered scale used to assess anxiety symptom severity [25]. The total score range is 0 to 56, where < 17 is indicative of mild severity; 18–24 mild to moderate severity; 25–30 moderate to severe; and > 30 very severe anxiety. The HAM-A has been shown to have sufficient reliability and concurrent validity [26].

#### ADHD symptom severity

The Adult Attention-deficit hyperactivity disorder Self-Report Scale (ASRS)-v1.1 Symptom Checklist [27] assesses the frequency of ADHD symptoms in adults over the preceding six months. The 18-item self-report questionnaire comprises three subscales: inattentive, motor hyperactive/impulsive and verbal hyperactive/impulsive [28]. The scale has high internal consistency (Cronbach’s alpha = 0.88) and concurrent validity (r = 0.84) [29], indicating good ability to evaluate ADHD for adults. When item-ratings are dichotomised the total score range is 0 to 18, with a score of ≥ 9 indicating elevated ADHD symptomology [27].

#### Quality of life

The self-reported WHO Quality of Life-BREF is a shortened form of the WHO Quality of Life-100 assessment [30] and is commonly used in epidemiological studies and clinical trials. It has 26 items that measure quality of life relating to physical health, psychological health, social relationships, and the environment over the past two weeks. Measures within the psychological domain include self-image, negative thoughts, positive attitudes, self-esteem, mentality, learning ability, memory, and concentration. Psychometric evaluation suggests that overall it is a sound, cross-culturally valid assessment of quality of life [31].

### Statistical analyses

Statistical analyses were designed to address 3 exploratory questions: (1) What are the effects of LDX on eating disorder psychopathology, depression, anxiety and ADHD symptoms, and psychological quality of life? (2) Are changes in BE frequency associated with concomitant changes in eating disorder psychopathology, depression, anxiety, ADHD and psychological quality of life? and (3) Do baseline levels of eating disorder psychopathology, depression, anxiety, ADHD and psychological quality of life impact the degree to which LDX alters BE frequency?

To evaluate the effects of LDX on each of the clinical measures of interest (Question 1), linear mixed models were performed for each measure. The clinical measure was entered as the dependent variable, individual as a random effect, and timepoint (Week 0, Week 8) as a fixed effect. To describe change at an individual-level (rather than the group mean), the proportion of individuals experiencing an increase or decrease in each measure was reported. This was determined by subtracting scores at time two from time one.

To determine whether concomitant changes occurred between clinical measures and BE frequency (Question 2), an interaction term was included in the timepoint model (change in clinical measure x timepoint), with BE frequency as the dependent variable. BE frequency was log-transformed to reduce skewness (number of binge eating days per week + 1) [6]. Simple effects analyses were used to follow up significant interactions. To plot the moderating effect of the measure on BE frequency, we used the mean value of the measure as well as one standard deviation above and below the mean value timepoints [32]. Analyses to address questions 1 and 2 were conducted both with and without baseline symptom severity scores as a covariate.

Linear mixed models with BE frequency as the dependent variable and a baseline clinical measure x timepoint interaction term were used to determine whether severity of any clinical measures at baseline were related to LDX-related change in BE frequency (Question 3).

Supplementary analyses replicating the above were also conducted on secondary measures, which were the subscales of the EDE-Q and the ASRS, and BMI. Supplementary t-tests were also conducted comparing trial completers versus non-completers on each of the clinical measures to determine if there were any baseline clinical features that potentially contributed to LDX being less tolerated. Bonferonni corrections were applied to account for multiple comparisons for primary (*q* < 0.01) and supplementary analyses on additional measures (*q* < 0.004).

Analyses were performed using R 3.5.1 [33]. The lme4, lmerTest and emmeans packages were used for Linear Mixed Models and post-hoc tests [34–36]. Figures were produced using ggplot and ggcorset packages (https://cran.r-project.org/web/packages/ggcorset/index.html).

## Results

**Fig. 1:**
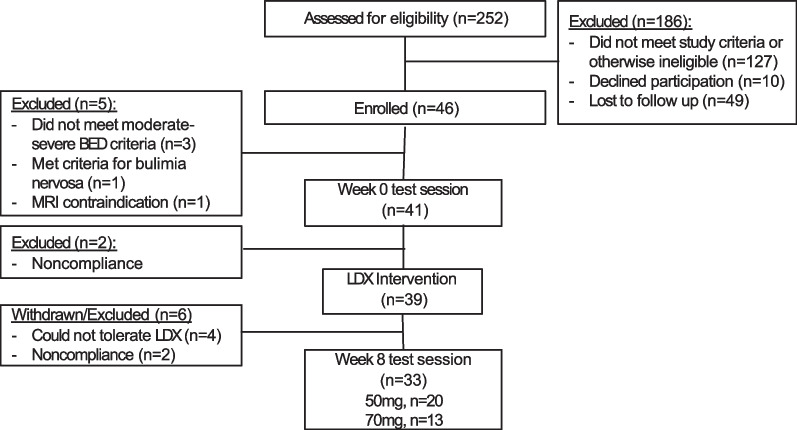
CONSORT participant flow diagram

Forty-one participants with BED were assessed at baseline (week 0) and 33 at medicated follow-up (week 8). The CONSORT diagram (Fig. 1), includes details of exclusions/withdrawals. Demographics and baseline clinical characteristics of participants are presented in Table 1. At study completion, 20 participants were taking 50 mg of LDX, while 13 were taking 70 mg. As reported previously from this sample [10], log BE frequency reduced from Week 0 (*M* = 0.70, *SD* 0.10; mean BE frequency 4.27/week) to Week 8 (*M* = 0.31, *SD* 0.20; mean BE frequency 1.33/week), [*t*(40.11) = −11.03, *p* < 0.001] (Cohen’s *d* effect size = 1.88).

**Table 1:** Demographics and clinical characteristics

	BED (n = 41)
Age, years M (*SD*)	26.6 (5.5)
Sex, n (%)	
Female	40 (97.6)
Race or ethnicity, n (%)	
Aboriginal and/or Torres Strait Islander	2 (4.9)
Asian	7 (17.1)
Caucasian	22 (53.7)
Hispanic	0 (0)
Other or multiple	10 (24.4)
Length of illness, years M (*SD*)	11.0 (7.6)
BMI category, n (%)	
Normal (19–25.0 kg/m^2^)	13 (31.7)
Overweight (≥ 25.0 to < 30.0 kg/m^2^)	16 (39.0)
Obesity Class I (≥ 30.0 to < 35.0 kg/m^2^)	8 (19.5)
Obesity class II (≥ 35.0 to < 40.0 kg/m^2^)	3 (7.3)
Obesity Class III (≥ 40.0 kg/m^2^)	1 (2.4)
Current psychiatric comorbidities, n (%)	
MDD	5 (12.2)
Anxiety	5 (12.2)
AUD/SUD	7 (17.1)
ADHD	5 (12.2)
Concurrent medication (class), n (%)	
SSRI	4 (9.8)
Anticonvulsant	2 (4.9)
Anxiolytic	2 (4.9)
Anti-diabetic	2 (4.9)
Proton-pump inhibitor	1 (2.4)
Diuretic	1 (2.4)
Thyroxine	1 (2.4)
Contraceptive	11 (26.8)

*M* mean, *SD* standard deviation, *n* number, *BMI* body mass index, *MDD* major depressive disorder, *AUD* alcohol use disorder, *SUD* substance use disorder, *ADHD* attention deficit hyperactivity disorder

*Anxiety includes Generalised Anxiety Disorder, Social Anxiety and Panic Disorder

Eight participants (19.5%) tested at baseline did not complete the trial. Of these, two did not commence LDX (one withdrawn due to metallic hair clips making her ineligible to participate in the MRI component and 1 due to lost contact). Four participants withdrew from the study due to experiencing adverse events. These were listed as increased symptoms of anxiety (*n* = 3), depression (*n* = 1), cognitive impairment (*n* = 1), and heartburn (*n* = 1). The remaining two were withdrawn due to lost contact/an inability to schedule a follow up test session (Fig. 1).

### How does LDX affect eating disorder psychopathology, depression, anxiety and ADHD symptoms, and psychological quality of life?

After 8 weeks of LDX, participants on average reported reduced eating disorder psychopathology (EDE-Q Global), *t*(35.20) = −10.77, *p* < 0.001 and increased psychological quality of life (WHOQOL Psychological),* t*(32.24) = 6.52, *p* < 0.001. There were no significant group level changes in depression severity (HAM-D) scores, *t*(32.82) = −1.95, *p* = 0.055, anxiety (HAM-A), *t*(28.82) = −0.43, *p* = 0.671, or ADHD (ASRS) severity, *t*(32.86) = −0.21, *p* = 0.836. See Table 2.

**Table 2:** Group-level change in clinical measures pre- post 8 weeks of Lisdexamfetamine

Measure (score range)	Wk 0 (*n* = 41)		Wk 0 (*n* = 33)		Wk 8 (*n* = 33)		Change, Cohen’s D ES, (95% CI)
	*M*	*SD*	*M*	*SD*	*M*	*SD*	
BE frequency (days/week) (0–7)**	4.17	1.22	4.27	1.18	1.33	1.05	1.88 (1.3–2.4)
EDE-Q global (0–6)**	4.64	1.03	4.60	1.03	2.92	1.09	1.60 (1.1–2.2)
HAM-D (0–54)	5.41	4.29	4.70	3.83	3.52	4.64	0.28 (-0.1 – 0.6)
HAM-A (0–56)	8.02	7.02	7.03	5.13	6.73	6.77	0.05 (−0.3 to 0.4)
ASRS total (0–18)	7.83	5.45	7.09	5.16	7.03	5.52	0.02 (−0.3 to 0.4)
WHOQOL-BREF psychological (0–100)**	46.14	14.89	47.00	14.05	59.38	12.88	0.93 (−1.34 to −0.51)

Mean (M), standard deviation (SD), effect sizes (ES) and confidence intervals (CI) reported

NB. Effect sizes and confidence intervals are calculated based on completer sample only, *n* = 33

*p < .01, **p < .001

**Fig. 2:**
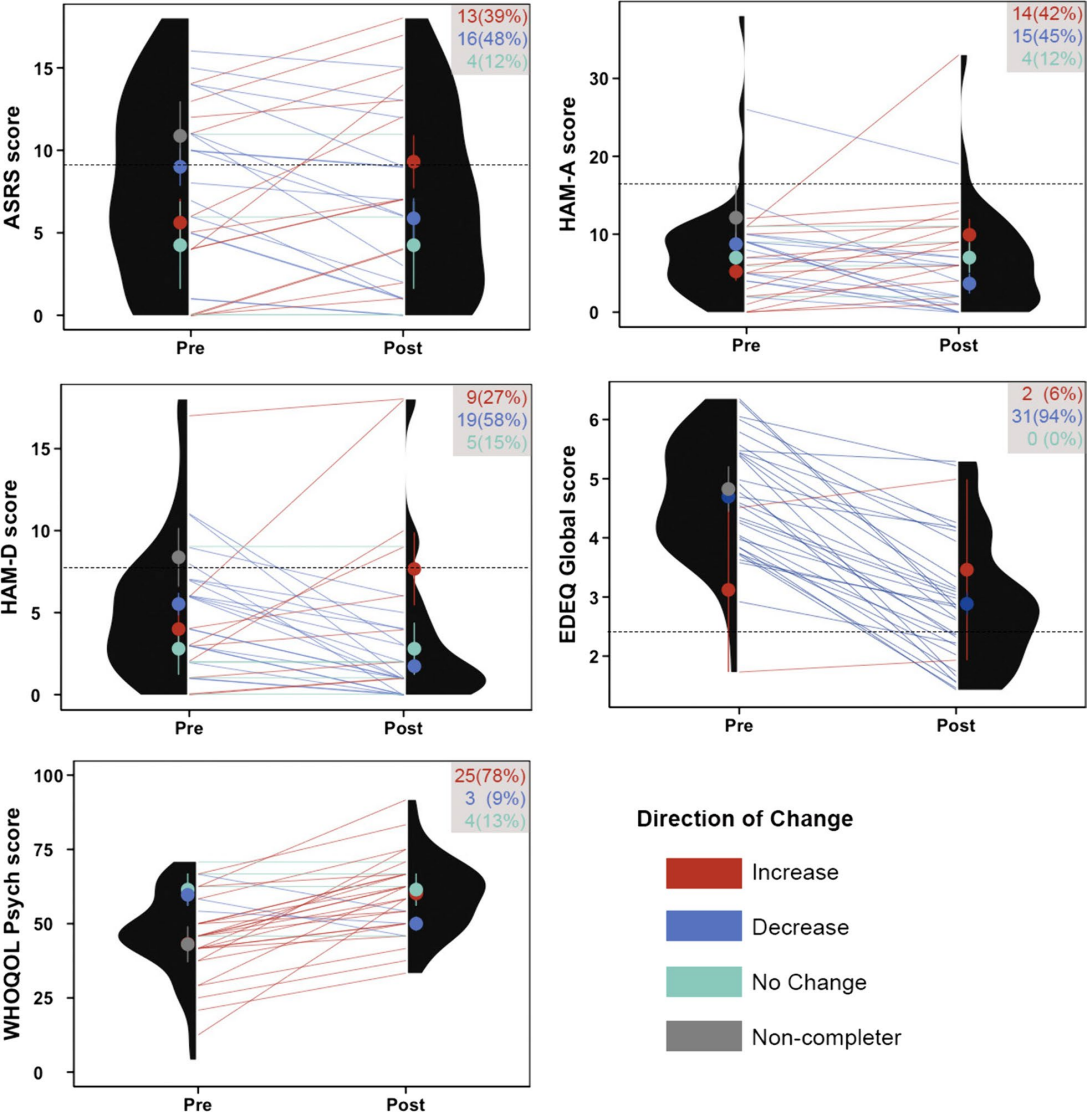
Corset plots showing individual changes and distributions of each of the primary outcome measures pre- and post-treatment with LDX. Colored circles and error bars show the mean and standard error from the mean of individuals who experienced an increase (red), decrease (blue) or no change (green) in symptoms. The grey circle in the pre- column indicates the mean and SEM of trial non-completers. Black dashed lines indicate the cut off at which symptom levels may be considered above normative levels. Colour-coded text in the grey insets indicate the frequency and proportion of individuals reporting increases, decreases or no change in each measure. ASRS, Attention Deficit Hyperactivity Disorder Adult Self-Report Scale; HAM-A, Hamilton Anxiety scale; HAM-D, Hamilton Depression Rating Scale; EDEQ, Eating Disorders Examination Questionnaire; WHOQOL Psych, World Health Organisation Quality of Life Psychological Subscale

Figure 2 illustrates the individual changes and distributions of each of the primary outcome measures pre- and post-treatment with LDX. The grey inset in the top right corner of each panel reports the frequency and percentage of individuals experiencing an increase, decrease or no change in each measure.

Due to low levels of co-occurring symptom severity across the group, post-hoc analyses were completed to examine change in depression, anxiety and ADHD symptom severity in the subset who reported above normative levels, according to the cut-offs provided by the instruments used. Nine participants (22%) reported mild-moderate depression. Of these individuals, 3 did not complete the trial, for reasons reported as unrelated to depression. Within the remaining 6 participants, there was a trend for a reduction in depression severity, (T1, *M* = 11.67, *SD* = 3.81; T2, *M* = 6.16, *SD* = 6.68), *t*(5.98) = −2.78, *p* = 0.033. These means were based on 4 (66%) experiencing decreases in symptom severity, 1(17%) remaining the same, and 1 (17%) experiencing an increase in depressive symptoms.

Three participants (7%) reported greater than mild anxiety (1 mild- moderate, 1 moderate-severe, and 1 very severe). Of these individuals, 2 did not complete the trial (1 due to increased agitation/anxiety, 1 unable to schedule a follow up session), and the remaining person experienced a small reduction in anxiety (26–19).

Eighteen participants (44%) reported elevated ADHD symptoms, based on the ASRS cut off of 9. Of these individuals, 4 did not complete the trial (2 due to anxiety/agitation, 1 unable to schedule a follow up and 1 withdrawn prior to medication commencement).

Within the remaining 14 participants, there was no change in total ADHD severity, *t*(14.34) = −1.69, *p* = 0.113, however there was a trend for a reduction in inattentive symptom severity, (T1, *M* = 8.06, *SD* = 1.0; T2, *M* = 6.64, *SD* = 2.17), *t*(16.84) = −2.62,* p* = 0.018. These means are based on seven participants (50%) experiencing reductions in inattentive symptom severity, 5 (36%) remaining unchanged and 2 (14%) experiencing increases.

### Are changes in BE frequency associated with concomitant changes in eating disorder psychopathology, depression, anxiety, ADHD and psychological quality of life?

There were no significant interactions between change in log BE frequency and change in EDE-Q Global, *t*(62) = 0.87, *p* = 0.390, HAM-D, *t*(34.67) = 0.927, *p* = 0.360, ASRS Total, *t*(30.99) = 1.35, *p* = 0.187 or WHOQOL Psychological, *t*(60) = −0.93, *p* = 0.355.

**Fig. 3:**
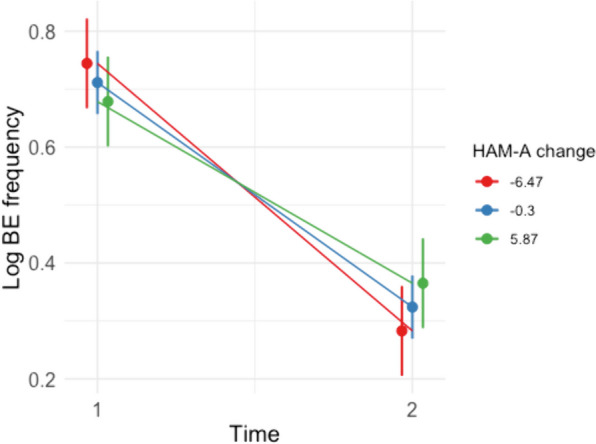
Plot showing significant interaction between change in log Binge Eating Frequency and anxiety (Hamilton Anxiety Rating Scale) from time 1 to 2. Following [32], we used the mean value of the moderator (i.e. change in verbal impulsivity) as well as one standard deviation above and below the mean value to plot the moderating effect of this measure on BE frequency between time points

There was a trend-level interaction between change in log BE frequency and change in HAM-A, *t*(30.99) = 1.94, *p* = 0.061, which remained unchanged when covarying for baseline HAM-A scores, *t*(61) = 1.95, *p* = 0.056. Follow up simple effects analysis showed that reductions in log BE frequency from Week 0 to Week 8 were most prominent for individuals with a reduction in HAM-A (i.e. around −6.47, *b* = 0.48, *t*(30.5) = 9.17, *p* < 0.001), and no change in HAM-A (i.e. around −0.25, *b* = 0.40, *t*(30.4) = 10.63, *p* < 0.001), and less prominent in those with increases in HAM-A (i.e. around 5.97, *b* = 0.31, *t*(29.9) = 5.81, *p* < 0.001; Fig. 3).

Post-hoc analyses were conducted to determine whether a common side effect of LDX, weight loss, also affected the clinical variables of interest. Average BMI significantly reduced from baseline (*M* = 27.79) to follow up (*M* = 26.33), *t*(32) = 8.33, *p* < 0.001. There was a significant interaction between change in BMI and change in psychological quality of life, *t*(30.78) = −2.96, *p* = 0.006, with greater BMI reductions associated with greater improvements in psychological quality of life. There was also a trend level interaction with change in eating disorder psychopathology, *t*(31.00) = 1.77, *p* = 0.09. Specifically, change in BMI interacted with change in shape concern, *t*(31.00) = 3.06, *p* = 0.005, and change in weight concern, *t*(31.00) = 2.23, *p* = 0.033, with greater reductions in BMI associated with greater reductions in shape and weight concerns.

To explore whether this was driven primarily by participants with higher baseline BMI, analyses were conducted on median-split derived groups: those with baseline BMI $\le$ 27 (n = 27) and in those with a BMI > 27 (n = 14). For the baseline BMI > 27 group, significant interactions remain between change in BMI and psychological quality of life *t*(9.00) = −2.28, *p* = 0.04, change in shape concern *t*(9.00) = 3.63, *p* = 0.005 and change in weight concern *t*(9.00) = 3.68, *p* = 0.005, whereas they were not significant for the BMI $\le$ 27 group.

### Does eating disorder psychopathology, depression, anxiety, ADHD and psychological quality of life at baseline impact the degree to which LDX alters BE frequency?

Change in BE frequency did not interact significantly with baseline EDE-Q Global (*t*(68) = 1.25, *p* = 0.214), HAM-D (*t*(68) = 1.28, *p* = 0.204), HAM-A (*t*(68) = 0.89, *p* = 0.377), ASRS (*t*(68) = −0.02, *p* = 0.981 or WHO-QOL Psychological (*t*(68) = −1.09, *p* = 0.280) scores.

Overall, 18/33 (55%) individuals achieved clinically-relevant treatment response (> 70% reduction in binge days per week). For individuals with diagnoses of comorbid current MDD (n = 4), 50% responded, with comorbid anxiety (n = 2), 50% responded, with comorbid ADHD (n = 4), 75% responded and with comorbid substance and alcohol use disorders (n = 3), 33% responded.

## Discussion

This study aimed to improve our understanding of how LDX affects not only eating disorder symptomology, but also mental health more broadly, and also to identify any clinical profiles that may predict better efficacy of LDX. After eight weeks of LDX, trial participants reported significantly improved psychological well-being and a reduction in their eating disorder psychopathology. A small majority of the subset of individuals reporting elevated ADHD and depressive symptoms at baseline experienced reductions in inattentive and depressive symptom severity. While there were no significant group-level reductions in anxiety, there was a trend for individuals with larger reductions in anxiety severity to concurrently report larger reductions in BE frequency. Finally, baseline levels of depression, anxiety, psychological well-being, or eating disorder psychopathology were not able to predict who would experience the largest reduction in BE frequency during eight weeks of LDX. This study provides proof of concept evidence that LDX is effective at improving symptoms of eating disorder psychopathology and psychological quality of life, and potentially ADHD and depression, in individuals with BED. Trial non-completers did not differ significantly to completers at baseline on any of these measures, however some participants withdrew due to increased anxiety.

This was the first clinical trial of LDX in moderate to severe BED that did not exclude participants with co-occurring psychiatric disorders. Despite this, the prevalence of *current* comorbidities and severity in the sample remained relatively low. With the group means of co-occurring symptom severity falling within the normative range, this floor effect left little opportunity for reductions. Importantly however, when examining the small subset with elevated depressive scores, the majority experienced reductions in depression. This mirrors findings reported in trials using LDX as an adjunct to antidepressant therapy [14], highlighting that LDX may potentially have mild antidepressant effects. It may also be a secondary effect due to reduction in binge eating frequency and the associated negative affect. Despite this clinical explanation for how change in BE frequency and depression severity may be linked, the current data is unable to support the hypothesis of a linear relationship in change between these measures. Our data also did not support a link between reduction in depression severity and BMI. It is promising however that the effects of LDX may extend beyond simple changes in eating behaviours for some individuals.

LDX has demonstrated efficacy for ameliorating symptoms of inattention and impulsivity, and is routinely prescribed for the management of ADHD [37]. Together with previous findings that LDX reduces impulsivity (as measured by the Barratt Impulsiveness Scale (BIS)) in people with BED [10, 11], we had anticipated significant reductions in ADHD symptom severity. There was a tentative finding of inattentive symptoms being reduced by LDX in those reporting elevated levels of ADHD symptoms at baseline, however there were no changes in motor or verbal impulsivity. Those reporting greater reductions in verbal impulsivity also experienced the greatest reduction in BE frequency. This subscale measures difficulty in controlling how much they talk, interrupt others, and in waiting their turn [28]. It may be that this construct somewhat maps onto the cognitively-driven behavioural control required to reduce binge episodes.

The discrepancy in LDX producing reductions in impulsivity measured by the BIS but not the ASRS may be accounted for by differences in aspects of impulsivity captured by these measures. For example, the BIS motor subscale items relate to acting without thinking while the motor impulsivity scale of the ASRS has items relating to an inability to sit still or relax, which relate more to hyperactivity and conceivably may be driven by anxiety. Similarly, the non-planning impulsivity subscale of the BIS assesses a lack of future orientation or forethought, which is not a DSM criterion for ADHD and therefore not assessed by the ASRS.

It is important to note that stimulant use in the past 6 months was an exclusion criterion for study entry, which may also have inadvertently biased our sample to milder cases. Overall, the results suggest that while LDX is not superior at reducing BE frequency in people with elevated symptoms of ADHD relative to those with no ADHD symptoms, in some individuals it will have the benefit of being able to improve symptoms of inattention alongside BE.

Given that a number of potential side effects of LDX mimic symptoms of anxiety, such as dry mouth, irritability, and increased heart rate, it is understandable that this medication may not necessarily reduce reported anxiety levels, particularly in highly anxious individuals. However, LDX also reduces the drive to binge eat, which for some individuals may resolve a significant source of anxiety. In the current sample, there were an equal proportion of people who experienced a decrease or an increase in anxiety (Fig. 2). There was a trend for those with the greatest reductions in anxiety to also report the greatest reduction in BE frequency. This supports previous accounts of the close relationship between anxiety and binge eating [38]. There are three potential explanations for this relationship; firstly, as stated above, a reduction in the drive to binge eat may alleviate anxiety. Second, there is consensus that high anxiety states may trigger binge eating episodes for some individuals, therefore a direct effect of LDX on reducing anxiety may produce its effect on reducing binge episodes. Finally, it is possible that both are mediated by a third unknown factor. As this medication is not known for its action on symptoms of anxiety, the second explanation seems less likely. A recent study examining the effect of stimulant medication in children with ADHD and co-occurring anxiety disorders found them relatively safe with regards to the risk of exacerbating symptoms of anxiety [39]. However heightened anxiety was provided as a reason for study withdrawal for three individuals, highlighting that anxiety should be closely monitored in people being prescribed LDX for BED.

We had anticipated that those with elevated symptoms of ADHD might experience the greatest reduction in BE frequency with LDX treatment due to its dual action on symptoms of ADHD and BED. Unfortunately, neither ADHD symptom severity, nor any of the other of the assessed clinical measures at baseline were able to predict who would respond best to LDX treatment in terms of reduction in BE frequency. Nevertheless, LDX does address both BE frequency and inattention for half of those with elevated ADHD symptoms, making it worthwhile for these individuals in their global presentation. Our previous work in this sample found that higher loss of control over eating and non-planning impulsivity at baseline predicted the best response to LDX [10]. The BIS-11 and Loss of Control Over Eating Scale (LOCES) remain the most promising means for clinicians to determine who may experience the greatest reductions in BE frequency from LDX.

An important clinical take away from this study was the robust improvement in eating disorder psychopathology and psychological quality of life. The strength of this broader improvement to mental health was unexpected given the lack of psychological intervention. These changes were not associated with the degree of reduction in BE frequency, showing that it is likely the gestalt of changes in a myriad of mental health conditions that lead to improved psychological quality of life. In addition, the degree of improvement in psychological quality of life and reduction in shape and weight concern (subscales from the EDE-Q) was associated with the amount of weight loss experienced across the trial, specifically in those of higher body weight at baseline. Many individuals with BED and higher body weight experience strong body dissatisfaction and wish to lose weight [40]. It may be that weight loss alone improves mental health [41], particularly if it alleviates any physical health concerns. It may also be that a reduction in weight yields a decrease in feelings and experiences of weight stigma and shame associated with higher body weight [42]. This relationship, and any mediators, requires further investigation. From a clinical perspective, it is important to monitor the influence of BMI change on self-reported quality of life and to ensure that this does not lead to disordered restrictive eating behaviours.

Of the six individuals who withdrew from the trial after commencing LDX, two entered the study with elevated levels of depression, anxiety and ADHD. LDX is likely less tolerable for highly complex presentations and psychological support should always be recommended.

This study has several limitations which must be taken into consideration. Firstly, this trial was designed to assess the mechanisms of action of LDX rather than its efficacy. As such, it was open-label and does not have a placebo control comparison. This does however replicate real-world settings, where placebo effects are part of the treatment experience. It was also conducted over a relatively short time frame, and future studies will need to examine if long term changes are maintained. Secondly, there was a lack of consistency across the instruments used to assess the severity of co-occurring conditions. The HAM-A and HAM-D are clinician-assessed while the WHO-QoL, EDE-Q and ASRS are self-report. There is mixed evidence about the level of agreement between self-report scales and clinician rating scales [43, 44], with an earlier meta-analysis suggesting that self-report measures are perhaps less sensitive to change than clinician rated scales for depression [45]. Further, the timescales across which symptoms were rated differed. For instance, the WHO-QoL, EDE-Q and ASRS ask about symptoms over the past two weeks, 28 days and six months, respectively. This may speak to how stable these measures are thought to be (state versus trait-based measures), but also means that the ASRS may not have had sufficient sensitivity to detect change over an 8-week trial period. Finally, in the current sample, there were relatively low severity levels of current co-occurring illness symptoms (although lifetime comorbidity rates were higher). As with previous trials of LDX in BED, exclusion requirements for safety purposes, such as hypertension and history of mania, may have made this sample less representative of a typical BED population. In addition, protocol requirements such as neuroimaging (for the broader study) may have deterred more anxious individuals from participating. Results found in the subgroups with elevated co-occurring symptoms rely on small sample sizes; it is important that this preliminary work is replicated in a larger sample with more significant levels of co-occurring illnesses and symptoms. Nonetheless, this study provides an important first step in understanding how co-occurring symptoms are affected by LDX in a more naturalistic study sample.

This study provides preliminary evidence that in people with BED, LDX may be effective at improving co-occurring symptoms of eating disorder psychopathology and psychological well-being, and potentially ADHD and depression symptoms when present at an elevated level. The impact on anxiety is more variable and should be monitored closely by clinicians. Overall, early indications suggest that it may be effectively used in people with BED, both with and without co-occurring psychiatric conditions, however this must be verified in larger, more varied samples.

### Supplementary Information

Additional file1 (DOCX 1077 kb)

## Data Availability

Data will be made available upon reasonable request to the corresponding author.
