# Supplementary material for: Exploring bi-directional impacts of Lisdexamfetamine dimesylate on psychological comorbidities and quality of life in people with Binge Eating Disorder
Source: J Eat Disord. 2024 Jun 13;12:80. doi: 10.1186/s40337-024-01041-9 (PMC11170888; doi:10.1186/s40337-024-01041-9)
Supplement: Supplementary file 1 — Additional file1 (DOCX 1077 kb) [file 40337_2024_1041_MOESM1_ESM.docx]

Exploring bi-directional impacts of Lisdexamfetamine dimesylate on psychological comorbidities and quality of life in people with Binge Eating Disorder.

Griffiths, K., Boulet, S., Barakat, S., Touyz, S., Hay, P.^4^ Maguire, S. and Kohn M.R.

**Supplementary Materials**

1. **Analyses of secondary measures (i.e. subscales and BMI)**

i) There were significant reductions in the EDE-Q subscales of eating concern (*t*(37.44) = -10.60, *p*<.001), shape concern (*t*(34.02) = -9.08, *p*<.001) and weight concern (*t*(33.82) = -7.84, *p*<.001), but not restraint (*t*(36.40) = -1.81, *p*=.079). There were no changes in Inattentive, *t*(33.66) = -1.15, *p*=.257, Motor Impulsivity, *t*(30.70) = 1.02, *p*=.318, Verbal Impulsivity, *t*(34.74) = -0.22, *p*=.825, subscales. BMI significantly reduced from baseline to follow up, *t*(31.96) = -8.18, *p*<.001.

ii) There were no significant interactions between change in log BE frequency and changes in Motor Impulsivity, *t*(62) = 0.59, *p*=.560, Inattention, *t*(62) = 0.49, *p*=.620, Restraint, *t(*62) = 0.40*, p*=0.688, Eating Concern, *t*(62) = 0.69, *p*=0.496, Shape Concern, *t*(62) = 0.91, *p*=.369, Weight Concern, *t*(62) = 0.57, *p*=.570 or BMI, *t*(30.99) = 1.24, *p*=.223.

There was a significant interaction between change in log BE frequency and change in Verbal Impulsivity, *t*(30.99) = 3.25, *p*=.003. Reductions in log BE frequency were most pronounced for individuals with reduced Verbal Impulsivity (i.e. around -1.08, *b*=0.50, *t*(31) = 10.20, *p*<.001) and no change in Verbal Impulsivity (i.e. 0.00, *b*=0.39, *t*(31) = 11.20, p<.001) and less pronounced in those with increases in Verbal Impulsivity (i.e. around 1.08, *b*=0.27, *t*(31) = 5.59, *p*<.001.


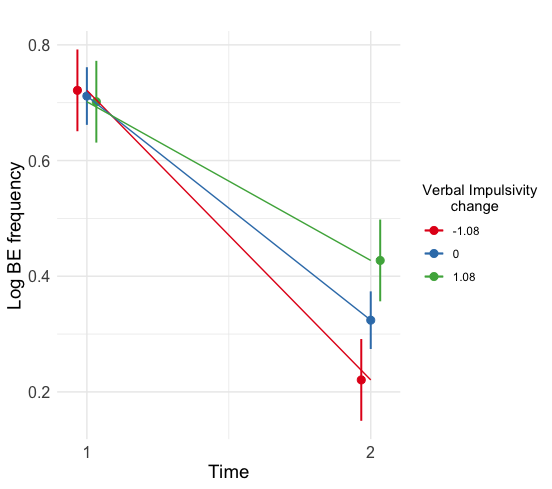


**Supplementary Figure 1.** Plot showing significant interaction between change in log Binge Eating Frequency and Verbal Impulsivity (ADHD Self-report Rating Scale subscale) from time 1 to 2. Following ^31^, we used the mean value of the moderator (i.e. change in verbal impulsivity) as well as one standard deviation above and below the mean value to plot the moderating effect of this measure on BE frequency between timepoints.

iii) Change in BE frequency did not interact significantly with baseline EDE-Q Shape Concern (*t*(70) = 1.07, *p*=.287), Weight Concern (*t*(41.97) = 1.38, *p*=.176), Eating Concern (*t*(70) = 0.59, *p*=.559), Restraint (*t*(70) = 0.16, *p*=.871, BMI (*t*(68) = -1.09, *p*=.280) scores. It also did not interact significantly with baseline Inattention, t(70) = -0.41, p=.682, Motor Impulsivity, t(70) = 0.16, p=.871, Verbal Impulsivity, t(70) = -0.51, p=.612 or BMI, t(62) = -0.44, p=.660.

**Table 1. Group-level change in clinical measure subscales and BMI pre- post 8 weeks of Lisdexamfetamine. Mean (M), Standard Deviation (SD) and Effect Sizes (ES) reported.**

| Measure (score range) | Wk 0  (*n*=41) | | Wk 8  (*n*=33) | | Change, Cohen’s D ES |
| --- | --- | --- | --- | --- | --- |
|  | *M* | *SD* | *M* | *SD* |  |
| EDE-Q |  |  |  |  |  |
| Restraint | 3.03 | 1.61 | 2.48 | 1.18 | 0.39 |
| Shape Concern** | 5.72 | 1.19 | 3.59 | 1.5 | 1.57 |
| Weight Concern** | 5.15 | 1.24 | 3.38 | 1.38 | 1.35 |
| Eating Concern** | 4.67 | 1.20 | 2.22 | 0.93 | 2.28 |
| ASRS |  |  |  |  |  |
| Inattention | 4.65 | 3.46 | 3.97 | 3.26 | 0.20 |
| Motor Impulsivity | 1.44 | 1.66 | 1.45 | 1.5 | 0.75 |
| Verbal Impulsivity | 1.73 | 1.30 | 1.61 | 1.39 | 0.09 |
| BMI** | 27.83 | 5.35 | 26.33 | 4.69 | 0.30 |

**p*<.01, ***p*<.001.

1. **Differences between trial Completers versus Non-completers**

A Welches t-test comparing trial completers (n=33) versus non-completers who commenced LDX (n=6) was conducted on each of the baseline clinical measures. There were no differences between trial completers and non-completers in depression, *t*(5.85) = -1.84, *p*=0.11, anxiety, *t*(5.31) = -1.5 *p*=.19, eating disorder psychopathology, *t*(6.40) = -0.04, *p*=.97, BMI, *t*(5.45) = 0.03 *p*=.98, or Log BE frequency, *t(*4.67) = 0.88, *p*=.42.
